# Global patterns of hemophilia drug trials, hemophilia care, and health care measures

**DOI:** 10.1016/j.rpth.2025.102714

**Published:** 2025-02-27

**Authors:** Stacey A. Fedewa, Leonard A. Valentino, Andee Koo, Lorraine Cafuir, Theresa W. Gillespie, Tyler W. Buckner, Duc Q. Tran, Ana Antun, Christine L. Kempton

**Affiliations:** 1 Department of Hematology and Medical Oncology, Emory University School of Medicine, Atlanta, Georgia, USA; 2 Hemophilia of Georgia Center for Bleeding and Clotting Disorders of Emory, Emory University School of Medicine, Atlanta, Georgia, USA; 3 Hemophilia and Thrombophilia Center, Rush University Medical Center, Chicago, Illinois, USA; 4 Division of Hematology, University of Colorado School of Medicine, Aurora, Colorado, USA; 5 Hemophilia of Georgia, Atlanta, Georgia, USA

**Keywords:** clinical trials, global health, health equity, hemophilia

## Abstract

**Background:**

Drug trials are vital to establish safe and effective treatments for congenital hemophilia, a bleeding disorder that affects about 800,000 males worldwide. The global distribution of hemophilia drug trials (HDTs) and their alignment with hemophilia care is unknown.

**Objectives:**

This study aimed to evaluate the global distribution of HDTs and its association with hemophilia care.

**Methods:**

In this cross-sectional study, HDTs conducted between 2007 and 2022 were selected from the clinicaltrials.gov database. The density of trials per 1000 expected males with hemophilia (eMwH) was assessed according to hemophilia care measures (factor VIII and IX utilization per 1000 eMwH) derived from World Federation of Hemophilia data.

**Results:**

Among 124 trials, 55 countries were represented, with an average of 7.9 countries per trial. Most HDT sites were in high-income (74.4%) or upper middle (20.1%)–income countries. The number of sites in lower-middle–income countries doubled, from 12 in 2007-2011 to 30 in 2017-2022—a nonsignificant increase from 5.8% to 7.0% (*P* = .53). Factor utilization was substantially reduced in lower-middle (0.4 international units [IUs] per 1000 eMwH) and upper middle (2.8 IUs per 1000 eMwH) compared with high (6.8 IUs per 1000 eMwH) income countries. HDT density was moderately correlated with factor usage (*r* = 0.436; *P* ≤ .001).

**Conclusion:**

Most HDT sites were located in high-income countries, although a substantial proportion were in upper middle–income countries. A small but increasing number of trials were conducted in lower-middle–income countries, where factor usage is relatively low. This study provides evidence on the global distribution of HDT and raises questions regarding the generalizability, barriers, opportunities, and ethics of trials for a rare bleeding disorder.

## Essentials

• In this global study of 124 hemophilia drug trials, most trial sites were located in high-income countries, though a substantial proportion were in upper-middle-income countries.
• A small but increasing number of trials were conducted in lower-middle-income countries, where factor usage is relatively low.
• This study provides evidence on the global distribution of hemophilia drug trials and raises questions regarding the generalizability, barriers, opportunities, and ethics of trials for a rare bleeding disorder.

## Graphical abstract

**Figure:**
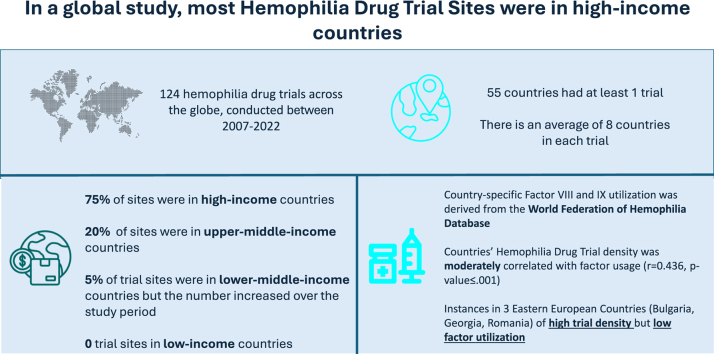

## 1 Introduction

Hemophilia is an X-linked blood clotting disorder in which people are deficient in factor (F)VIII (hemophilia A) or FIX (hemophilia B). This results in an increased risk of spontaneous bleeding, bleeding following injuries or surgery, and premature death. Drug trials for hemophilia have led to the approval of factor-replacement and non–factor-replacement therapies, which have reduced bleeding rates and improved the life expectancy and quality of life for those with access to hemophilia care [1,2]. Now that several effective hemophilia treatments have been developed, the hemophilia research, clinical, and advocacy communities have expanded their goals to ensure more equitable access to diagnosis and treatment worldwide [3,4]. To guide these efforts, the World Federation of Hemophilia (WFH) publishes an annual report with country-specific disease burden and factor usage per capita, and there are a growing number of hemophilia registries measuring global access to hemophilia care and outcomes [5,6].

However, there is a lack of information on the global distribution of hemophilia drug trials (HDTs). Understanding the global landscape of HDTs is an initial step in identifying potential inequities, opportunities, and barriers in hemophilia trial participation. Furthermore, identifying lack of equitable representation is important because it may limit trial generalizability and compound disparities in health outcomes, leading to poorer outcomes in underrepresented groups [7]. In this study, we had 2 primary aims: (1) to evaluate the global distribution of HDTs according to period, geographic region, and income category and (2) to examine whether trial density was associated with hemophilia care and health indicators.

## 2 Methods

In this cross-sectional observational study, we used several data sources summarized in Supplementary Table 1. First, the clinicaltrials.gov database was used to identify hemophilia clinical trials. The US National Institutes of Health launched the clinicaltrials.gov database in 2000 to collect data on trials with new drug applications, although its purpose has expanded over time [8]. Sponsors and investigators submit data to the repository because they are required to do so by laws/regulations or voluntarily. Beginning in 2005, the International Committee of Medical Journal Editors required authors to submit clinical data to clinicaltrials.gov, and in 2006, the World Health Organization (WHO) recommended that trials report data to clinicaltrials.gov or similar databases [8]. In 2007, the Food and Drug Administration expanded the types of studies required to submit data to the repository. Clinicaltrials.gov is the largest clinical trial database in the world, and it contains information on trials conducted outside of the US given the requirement of International Committee of Medical Journal Editors and WHO recommendation for sponsors to submit information to the registry [9]. We queried the clinicaltrials.gov database in April 2023 for trials with “hemophilia” as the condition, that were coded as “interventional” and with “results,” which resulted in 157 identified studies that were completed between 2007 and 2022. We further reviewed the identified trials to select those that tested drugs. The clinicaltrials.gov registry contains trial characteristics (phase, start and completion dates, sponsors, and trial site locations) and participant characteristics (participant age, race, ethnicity, and sex) as part of their data submission process. This study was deemed “non-human” subjects research under the Emory University Institutional Review Board.

The WFH annual global survey data were used to capture country-specific hemophilia care metrics, which included per-capita FVIII and FIX usage per country, and observed vs expected male cases of hemophilia [5]. The number of observed to expected cases is a marker of access to hemophilia care and diagnostic capacity. Factor utilization is a marker of access and delivery of hemophilia care. The WFH began sending annual questionnaires to national member organizations (NMOs) to capture information on demographics and hemophilia care in 1998. Data are self-reported by NMOs and are not verified, including factor usage unless the country participates in the WFH humanitarian program [10]. Historical data on factor utilization were available in the 2021 report, and we relied on factor data reported before the approval of emicizumab (before 2017) as the introduction of emicizumab in some countries may have led to a decline in factor usage. If a country’s factor data were not reported before 2017, more recent data (from 2017 to 2021) were used. The number of observed male cases reported by NMOs and the number of expected male hemophilia cases were estimated for each country using the following formula:

Expectednumberofmalehemophiliacases=MaleCensusPopulation×HemophiliaPrevalence

The expected number of male hemophilia cases was derived from census populations and the estimated global prevalence of disease, which is 17.1 and 3.8 per 100,000 males for hemophilia A and B, respectively [11]. Male population estimates were derived from the 2021 World Bank database. Estimates for female cases were not computed as there are no known published prevalence estimates for women and girls with hemophilia, and most clinical trials were conducted among males only.

The WHO data were used to measure health care and health indicators in 2021, which included each country’s physician density and level of total expenditure on health as a percentage of gross domestic product (GDP). Physician density was used to capture how well a country’s health system is functioning. Health spending as a portion of GDP was used to capture a country’s investment in its health system. Countries were coded into the following WHO 7 regions: African, Region of the Americas, South-East Asian, European, Eastern Mediterranean, and Western Pacific and 4 income classifications according to the World Bank: low-income countries (LICs), low-middle–income countries (LMICs), upper middle–income countries (UMICs), and high-income countries (HICs) (see Supplementary Table 2 for more information).

### 2.1 Statistical analyses

We first examined trial characteristics, including the phase, number of participants, number of countries represented, and geographic region. Next, we examined country-specific outcomes that included a country’s number of trials and density of clinical trials per 1000 expected male hemophilia population as follows:

$$\text{Density}\;\text{of}\;\text{clinical}\;\text{trials}=\frac{\text{Number}\;\text{of}\;\text{clinical}\;\text{trials}}{\text{Expected}\;\text{number}\;\text{of}\;\text{hemophilia}\;\text{cases}\;}x\;1000$$

Even if there were multiple sites within a country for a trial, the country was counted as being represented only once in the trial. The number of times a country was represented across trials was used to compute trial density.

Outcomes were assessed according to trial characteristics (phase and participant characteristics), WHO region, and income, as well as measures of hemophilia care (observed to expected cases, factor utilization), and general health care characteristics (physician density and health care spending as a proportion of GDP). Clinical trial characteristics were also assessed according to time period. These comparisons were made with chi-squared statistics and Pearson *r* correlation statistics.

## 3 Results

There were 157 trials completed between 2007 and 2022 in the clinicaltrials.gov registry that met the initial selection criteria; 28 trials were excluded because they were incomplete, and 5 trials were excluded because they examined response to hepatitis C treatment by people with hemophilia or were among people with acquired hemophilia. Among the 124 trials included in the final analyses, 42.7% were completed in 2017-2022, most trials were industry sponsored (>90%), over half were phase III (54.0%), and there was an average of 61.6 (SD, 55.2) participants per trial (Table 1). Overall, the average participant age was 26.4 years, and most trials included males only (95.2%). Approximately 61.3% and 23.4% of trials included participants with hemophilia A and hemophilia B, respectively, and 14.5% included both those with hemophilia A and B. Additionally, 21.0% of trials included participants with inhibitors (alloantibodies directed against FVIII or FIX), and most trials (>75%) were conducted among those with severe or moderate/severe hemophilia.

**Table 1:** Characteristics of hemophilia interventional clinical trials (2007-2022)^a^

Characteristic	No of trials, *n* (%)	Included LMIC and/or UMIC, *n* (%)	Included HIC only, *n* (%)	*P*
Total	124 (100)	98 (79.0)	26 (21.0)	
Study completion date				.005
2007-2012	35 (28.2)	22 (62.9)	13 (37.1)	
2013-2016	36 (29)	34 (94.4)	2 (5.6)	
2017-2022	53 (42.7)	42 (79.2)	11 (20.8)	
Phase				.006
I	12 (9.7)	7 (58.3)	5 (41.7)	
II	21 (16.9)	16 (76.2)	5 (23.8)	
III	67 (54)	61 (91)	6 (9)	
IV	22 (17.7)	14 (63.6)	8 (36.4)	
Missing	2 (1.6)	0 (0)	2 (100)	
Trial length (y)				.31
<2	46 (37.1)	38 (82.6)	8 (17.4)	
2-5	53 (42.7)	43 (81.1)	10 (18.9)	
>5	25 (20.2)	17 (68)	8 (32)	
Sponsor type				<.001
Industry	118 (95.2)	98 (83.1)	20 (16.9)	
Academic	6 (4.8)	0 (0)	6 (100)	
Hemophilia type				.06
Hemophilia A	76 (61.3)	57 (75)	19 (25)	
Hemophilia B	29 (23.4)	24 (82.8)	5 (17.2)	
Hemophilia A and B	18 (14.5)	17 (94.4)	1 (5.6)	
Missing	1 (0.8)	0 (0)	1 (100)	
Severity				.85
All severities^b^	10 (8.1)	9 (90)	1 (10)	
Moderate and severe	78 (62.9)	61 (78.2)	17 (21.8)	
Severe	18 (14.5)	14 (77.8)	4 (22.2)	
Missing	10 (8.1)	9 (90)	1 (10)	
Included inhibitor patients	26 (21)	22 (84.6)	4 (15.4)	.43
	Mean (SD)			
No. of countries	7.9 (6.1)	9.3 (5.9)	2.5 (2.7)	<.001
No. of Participants	61.6 (55.21)	69.2 (57.09)	33 (35.78)	.009
Participant age	26.4 (13.1)	25.6 (13.4)	30.0 (11.2)	.421

HIC, higher-income country; LMIC, lower-middle–income country; UMIC, upper middle–income country.

a *P* value compares LMIC/UMIC and HIC countries. Chi-squared tests were used to examine differences in categorical variables. A *T*-test was used to examine the age difference.

b Included mild, moderate, and severe. There were no trials with only moderate and only mild participants.

Trial size and phase were relatively consistent over time, although there was a significantly higher proportion of trials sponsored by academic intuitions during 2007-2012 (14.3%) vs 2013-2016 (0%) and 2017-2022 (1.9%; *P* = 0.008) (Supplementary Table 3). In terms of country income categories, there was a decreasing proportion of trials conducted solely in HIC. For example, 37.1% of trials in 2007-2011 were conducted in HIC only compared to 5.6% of trials in 2012-2016 and 20.8% of trials in 2017-2022 (*P* = .005) (Table 1). Trials conducted only in HIC had fewer participants and were more likely to be Phase I/II trials, compared to trials that included at least one LMIC or UMIC.

There were 55 countries with at least 1 clinical trial and an average of 7.9 countries represented per trial, which nonsignificantly increased over time from 6.3 in 2007-2011 to 9.2 and 8.1 in 2012-2016 and 2017-2022, respectively (*P* = .135). There were 960 unique country-trial sites during the study period, and on average, a country had an average of 2.15 trials per 1000 eMwH. Trial density was moderately correlated with FVIII and FIX usage (*r* = 0.436; *P* < .001) and physician density (*r* = 0.417; *P* = .001) and weakly correlated with the expected number of men with hemophilia and the amount a country spent on health care as a percentage of the GDP (Table 2).

**Table 2:** Correlation coefficients of country RCT density, factor utilization, hemophilia access, and general health care characteristics.

	RCT density	Factor utilization	Expected No. of men with hemophilia	Density of doctors	Health GDP
Characteristic	Pearson correlation coefficient (*P*)				
RCT density		0.436 (<.001)	−0.042 (.53)	0.417 (<.001)	0.261 (<.001)
Factor utilization	0.436 (<.001)		−0.074 (.41)	0.635 (<.001)	0.543 (<.001)
Expected No. of men with hemophilia	−0.042 (.53)	−0.074 (.41)		−0.051 (.498)	−0.103 (.17)
Density of doctors	0.417 (<.001)	0.635 (<.001)	−0.051 (.50)		0.519 (<.001)
Health GDP	0.261 (<.001)	0.543 (<.001)	−0.103 (.17)	0.519 (<.001)	

GDP, gross domestic product; RCT, randomized clinical trial.

**Figure 1:**
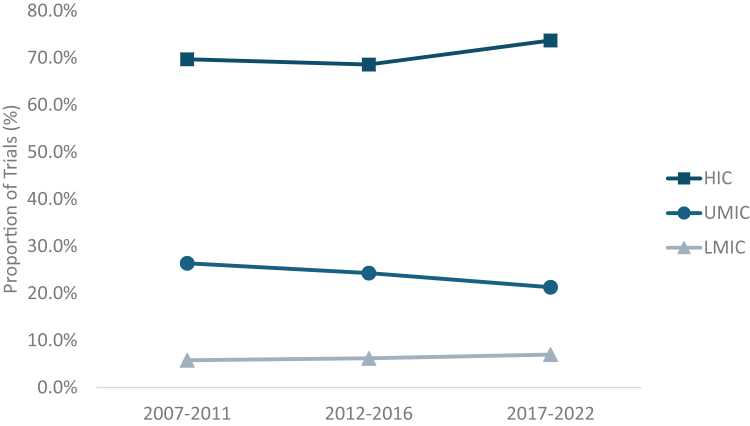
The proportion of hemophilia clinical trial sites according to World Bank Income Categories (2007-2022). HIC, higher-income country; LMIC, lower-middle–income country; UMIC, upper middle–income country. There were no trials conducted in low-income countries.

Among the 960 country-trial sites, nearly 75% were in HICs (74.4%), about 20% were in UMICs (20.1%), and 5.4% were in LMICs. There were no trials conducted in LICs. During the study period, there was a slight increase in the proportion of trial sites in HICs (from 69.7% in 2007-2011 to 73.7% in 2017-2022) and a decrease in sites in UMIC (from 26.4% in 2007-2011 to 21.3% in 2017-2022). The number of sites in LMICs was more than doubled from 12 to 30, and the proportion nonsignificantly increased from 5.8% in 2007-2011 to 7.0% in 2017-2022 (Figure 1). The findings of the number of sites per country versus the proportion of trials with at least 1 site in a country show that the number of clinical trials with at least 1 site in LIC and UMIC increased, the volume of sites in LMICs is somewhat parallel with that of HICs. Per capita FVIII and FIX use was substantially lower in LMICs (0.4 international units [IUs] per 1000 eMwH) and UMICs (2.8 IU per eMwH) compared with that in HICs (6.8 IUs per eMwH) (Supplementary Table 4).

**Figure 2:**
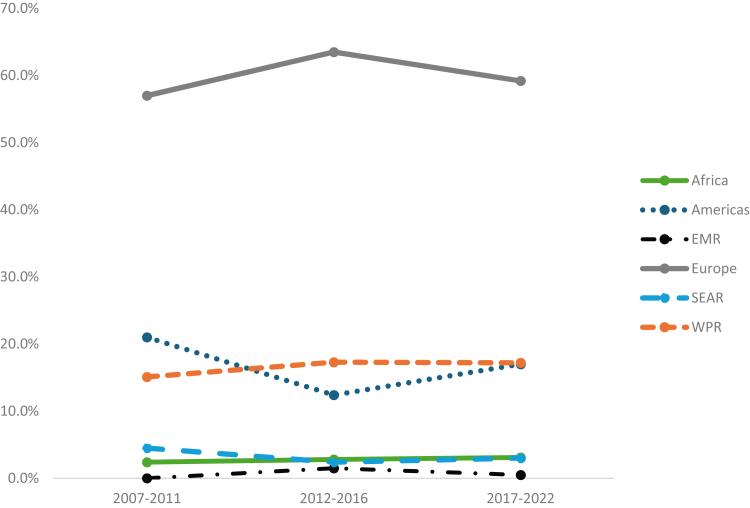
Proportion of hemophilia clinical trial sites according to World Health Organization global regions (2007-2022). EMR, Eastern Mediterranean Region; SEAR, South East Asian Region; WPR, Western Pacific Region.

**Figure 3:**
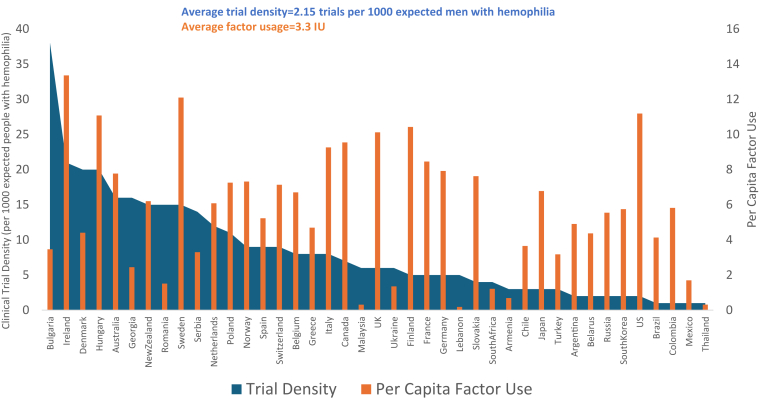
Randomized clinical trial density and factor utilization among 44 countries with trial and factor data.

In terms of WHO region, Europe was consistently the most common trial site location with 60.7% of trial sites during the entire study period (2007-2022), followed by the Americas (16.7%), Western Pacific (15.9%), Southeast Asia (3.3%), African (2.8%), and Eastern Mediterranean (0.7%) regions (Supplementary Table 5). The proportion of sites located in Africa, Europe, and the Western Pacific regions increased slightly over time (Figure 2). In terms of absolute numbers, the following countries were most represented in clinical trials: the United States (*n* = 81), Italy (*n* = 48), Australia (*n* = 43), Poland (*n* = 43), Germany (*n* = 41), the United Kingdom (*n* = 41), and Spain (*n* = 39) (Supplementary Figure 1). Regarding density, which accounts for underlying differences in population density, the most trial-dense countries were Bulgaria, Croatia, North Macedonia, Austria, Israel, and Ireland, with ≥20 trials per 1000 eMwH (Supplementary Figure 2). Bulgaria, Georgia, and Romania had far exceeded trial densities and had factor usage far lower than global averages (Figure 3).

## 4 Discussion

In our study of 124 HDTs completed between 2007 and 2022, most trial sites (75%) were located in HICs, although trials are increasingly being conducted globally. UMICs accounted for 20% of all trial sites, and while the number of trial sites in LMICs nearly doubled, they represented a small proportion (5%) of all trial sites. The density of clinical trials in a country was moderately correlated with factor usage, although there were outliers. For example, several Eastern European countries including Bulgaria, Georgia, and Romania had the highest per-capita trial density but relatively low factor usage. These countries have relatively large populations and adequate infrastructures to support clinical trials in large cities but the lack of coordinated medical services and the high cost of healthcare limit access to care for those with rare diseases like hemophilia [12,13].

Our finding that HDTs are increasingly multinational yet are mostly located in HICs is in line with broader trends of where clinical trials are occurring. In an analysis of 509 clinical trials across 25,000 sites, the proportion of multinational clinical trials almost doubled between 1995 and 2005, and there was an increasing number of trials in Africa and Eastern Europe [14]. In studies of common chronic diseases (chronic kidney disease and cancer), there are increases in LIC and LMIC trial sites, yet these countries remain underrepresented when compared with disease burden [15,16]. In terms of equitable global trial site selection, previous work has suggested that the number of clinical trial participants should be proportionate to regions where current treatments are being used. However, more recent thoughts on this issue have suggested that clinical trials should be conducted per population burden and where drugs are needed [14,17]. For hemophilia, it is unclear what equitable site selection would be given intercountry differences in terms of who is diagnosed with hemophilia according to severity, inhibitor status, and sex.

The lack of global representation of hemophilia may limit the trials’ generalizability if efficacy and safety end points vary across populations or if there are inherent genetic differences among populations. For hemophilia, the annualized bleeding rate is the most common primary end point, and while baseline factor levels are highly correlated with bleeding phenotype and are part of trial selection criteria, procoagulant and anticoagulant factors are known to influence bleeding risk and may vary globally and possibly by ethnicity [[18], [19], [20]]. While there are currently no known ethnic differences in the pharmacokinetics of factor-replacing therapies used to treat hemophilia, there are variations in factor clearance and half-life among people with hemophilia and environmental factors, as well as culture and ethnicity influence the pharmacokinetics of other medications [20,21]: for example, in occurrence of the HLA-B∗1502 allele and the incidence of adverse reactions like Stevens–Johnson Syndrome and toxic epidermal necrolysis following exposure to carbamazepine or the occurrence of multiple copies of the *CYP2D6* gene among Arab populations and the rapid metabolism of codeine to morphine resulting in higher levels of morphine in the blood [22]. Furthermore, environmental factors contribute to bleeding risk, including occupational injuries and accidents, which are higher in LICs and LMICs compared with those in HICs, which may influence annualized bleeding rates [23]. Thus, it is plausible that efficacy and safety end points could vary across populations or that different dosing strategies are needed for different populations.

To optimize the benefits of conducting hemophilia clinical trials globally, barriers need to be addressed to ensure that trials are feasible and can be conducted within a reasonable timeline. Broader barriers to clinical trials in developing countries include inadequate financial resources, limited health care and regulatory infrastructures, competing demands, and physician awareness of clinical trial availability [24,25]. As observed in our study, health care infrastructure (physician density) was modestly correlated with hemophilia trial density; however, general health care spending was not, which may indicate that health care infrastructure may play a larger role in hemophilia trial capacity compared with general health care spending. In addition to these general health care barriers, trials for hemophilia may face unique barriers given that it is a rare disease and is underdiagnosed in countries with limited resources. For example, it is estimated that <10% of people with hemophilia in Africa have been identified compared with 80% of people in Europe. Hemophilia clinical trials also require specialized coagulation laboratories and immediate processing of specimens, and hemophilia care is often centralized and requires participants to travel long distances [26]. For example, a study of 5 developing countries found that participants traveled an average of 80 km (or 52 miles) to receive hemophilia treatment, highlighting the need for compensation of research participants for their time [27]. Additionally, safe storage and transportation of a product, and support for treatment including self-infusion, may be barrier barriers and limit the use of mobile and digital technologies. Decentralized trials have certain features (noncentralized recruitment strategies) that may improve HDT participation in limited resource settings, but other aspects of decentralized trials, such as wearable technology and digital applications, are not as applicable to HDTs given the complexity of hemophilia treatment [28].

Despite the challenges, there is a small yet growing number of trials in LMICs. Additional research on what made these successful partnerships could be conducted, to inform future trial site selection. The WFH’s current outreach programs (treatment center twining, and the cornerstone initiative which provides support, expertise, and training to countries with minimal levels of care) may be used to better understand the unique barriers and facilitators of hemophilia trials. Other studies note that these countries have been attributed to lower costs due to less expensive staff salaries and logistics [29,30]. Sponsors may also be motivated to conduct clinical trials in LMIC to reach recruitment goals more rapidly as people in limited resource settings may be more willing to participate, and depending on the trial design, underresourced settings may have a greater number of eligible participants. For example, limited resource settings may contain more people with hemophilia who have not been previously treated with prophylaxis, which is a helpful group of people to study to improve understanding of the risk of inhibitor development [29]. Recruitment of more diverse settings may also improve trials’ racial and ethnic diversity, which is a goal of the US Food and Drug Administration and professional societies, including the American Society of Hematology [31]. Ensuring racial and ethnic diversity in hemophilia clinical trials is especially relevant considering that in our earlier work, <10% of participants in hemophilia clinical trials are Black or Hispanic [32]. We also observed that <2% of participants were women and girls, which is another equity issue to consider, especially in the context of global clinical trials, given that the number of women and girls diagnosed with hemophilia to 1 man with hemophilia, there are 2.7 to 5 potential carriers and 1.56 are somatic carriers [33]. This is particularly important, especially in the context of global clinical trials, given that the number of women and girls diagnosed with hemophilia is especially low in low-resource settings [34].

In addition to the barriers and facilitators of hemophilia clinical trials, there are also ethical questions regarding the globalization of hemophilia trials—especially given our observation that factor utilization varied across countries and that there were instances in Eastern Europe (Bulgaria, Georgia, and Romania) where trial density was high but factor usage was low. Previous studies have noted an increasing number of clinical trials in Eastern Europe as there is an existing clinical trial structure, higher patient recruitment, and lower costs, which may explain our finding of relatively high trial density in Bulgaria, Georgia, and Romania [35,36]. The lower-than-average factor utilization in these countries raises concerns that populations contributing to drug testing may not have adequate access to novel treatments and lack of available resources for clinical care may provide a void that research participation can fill. A review of this topic outlined the expectations of sponsors, including questions and decision guides for trials that are testing drugs for which the nation of testing may not benefit, noting the need to balance risks versus benefits [37]. A previous study of the availability of novel drugs for several diseases showed that among countries hosting clinical trials—market access was granted in 46% of HICs compared with <22% of UMICs and LMICs, indicating a lack of equitable distribution of research benefits at the population level [30,38] Furthermore, the cost for health systems and patients may be a major barrier as clinical trials provide access to therapies for many who otherwise would not be able to afford the commercialized product. We did not have information on whether trial participants had access to their treatments following the completion of the trial. The 2013 Declaration of Helsinki documents that trial sponsors, researchers, and governments should make plans for posttrial access to therapeutics for trial participants, but how frequently this guidance is followed for hemophilia trials is unknown [39]. Industry sponsors may also agree to provide compassionate use of products, although a recent study by a drug company across all therapeutic areas showed that most compassionate use requests were received most commonly from HICs and that barriers in LMICs remain [40]. Once licensed, sponsors could expand their role in the WFH humanitarian aid program, which has successfully scaled up and expanded product use to treat acute bleeds, facilitate surgeries, and increase prophylaxis use [10]. To support WFH’s goal of ensuring hemophilia treatment for all, the generalizability of global trials, and ethics, and barriers, while the balance of timeliness of approvals must be considered by all stakeholders, including patients, families, sponsors, regulatory agencies, health care systems, and public health/nonprofit programs.

There are several limitations to our study, including the inability to assess the number of participants per site or country. Thus, we were unable to assess country-level differences in participant volume. Additionally, the clinicaltrials.gov database may not capture all interventional trials during our study period, although, given the reporting and publishing requirements, we presumed that most practice-changing interventional trials are mostly represented. In targeted audits of the clinicaltrials.gov registry (that included all diseases), there was imperfect reporting compliance, although >65% of industry-led trials reported results to clinicaltrials.gov and nearly 100% of all trials led by larger pharmaceutical companies reported data [9]. Furthermore, trial locations could be miscoded; however, the Food and Drug Administration has used trial locations for their reporting purposes [8]. We were also unable to measure access to drugs following the trial completion, and we did not have information on country-specific market authorization or trends in uptake. To mitigate the role of emicizumab in a country’s factor usage, we used factor usage estimates to 2017, when emicizumab was initially approved. Despite these limitations, our study is the first to examine the global distribution of hemophilia clinical trials, informing global health equity and research efforts.

Hemophilia clinical trial sites were mostly located in HICs, although a substantial proportion were in UMICs and an increasing number of trials are being conducted in LMICs, where per-capita factor usage is a fraction of what is used in HICs. There were also several Eastern European countries with higher-than-average clinical trial density, with lower-than-average factor usage. These provide a baseline estimate to measure changes in global hemophilia trials. Furthermore, our findings raise questions regarding the barriers, opportunities, and ethics of global clinical trials, within the context of a rare disease.
