# Supplementary material for: Global patterns of hemophilia drug trials, hemophilia care, and health care measures
Source: Res Pract Thromb Haemost. 2025 Feb 27;9(2):102714. doi: 10.1016/j.rpth.2025.102714 (PMC11992419; doi:10.1016/j.rpth.2025.102714)
Supplement: Supplemental Material [file mmc1.docx]

Supplemental Figure 1. Number of Trials Sites Across the Globe


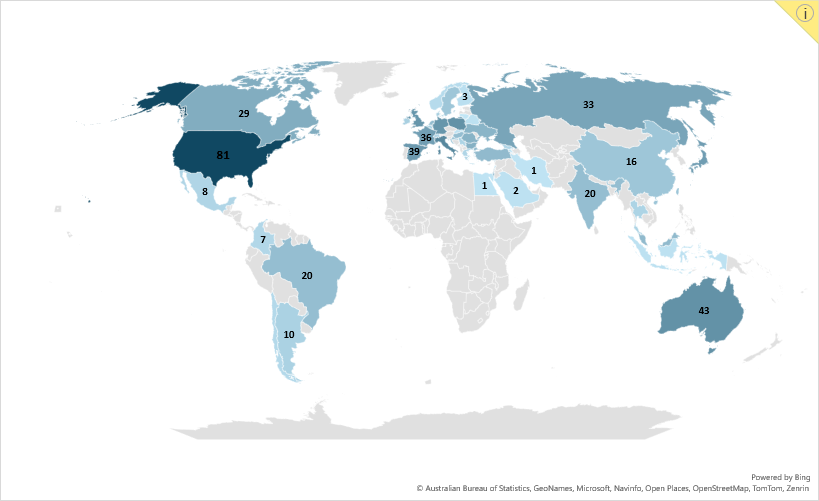


**Supplemental Figure 2. Clinical Trial Density per 1000 expected men with hemophilia**

**Supplemental Table 1. Data Source and Attributes**

| **Data Source** | **Type of data included** |
| --- | --- |
| Clinicaltrials.gov | Interventional clinical trial information |
| World Federation of Hemophilia (WFH) Annual Survey and Directory | Hemophilia care (number of HTCs, factor usage, expected and observed number of cases) |
| World Health Organization | Health indicators (research budget, physician density, vaccination, health to gross domestic product ratio, life expectancy) and regions |
| United Nations | Human development index |

**Supplemental Table 2. World Bank Classifications**

| **Regions/Income Classifications** | **Countries** |
| --- | --- |
| **African** | Algeria, Angola, Benin, Botswana, Burkina Faso, Burundi, Cameroon, Cape Verde, Central African Republic, Chad, Comoros, Ivory Coast, Democratic Republic of the Congo, Equatorial Guinea, Eritrea, Ethiopia, Gabon, Gambia, Ghana, Guinea, Guinea-Bissau, Kenya, Lesotho, Liberia, Madagascar, Malawi, Mali, Mauritania, Mauritius, Mozambique, Namibia, Niger, Nigeria, Republic of the Congo, Rwanda, São Tomé and Príncipe, Senegal, Seychelles, Sierra Leone, **South Africa**, South Sudan, Eswatini, Togo, Uganda, Tanzania, Zambia, Zimbabwe**.** |
| **Region of the Americas** | Antigua and Barbuda, Argentina, Bahamas, Barbados, Belize, Bolivia, Brazil, Canada, Chile, Colombia, Costa Rica, Cuba, Dominica, Dominican Republic, Ecuador, El Salvador, Grenada, Guatemala, Guyana, Haiti, Honduras, Jamaica, Mexico, Nicaragua, Panama, Paraguay, Peru, Saint Kitts and Nevis, Saint Lucia, Saint Vincent and the Grenadines, Suriname, Trinidad and Tobago, United States, Uruguay, Venezuela |
| **South-East Asian Region** | Bangladesh, Bhutan, North Korea, India, Indonesia, Maldives, Myanmar, Nepal, Sri Lanka, Thailand, Timor-Leste |
| **European** | Albania, Andorra, Armenia, Austria, Azerbaijan, Belarus, Belgium, Bosnia and Herzegovina, Bulgaria, Croatia, Cyprus, Czech Republic, Denmark, Estonia, Finland, France, Georgia, Germany, Greece, Hungary, Iceland, Ireland, Israel, Italy, Kazakhstan, Kyrgyzstan, Latvia, Lithuania, Luxembourg, Malta, Moldova, Monaco, Montenegro, Netherlands, North Macedonia, Norway, Poland, Portugal, Romania, Russia, San Marino, Serbia, Slovakia, Slovenia, Spain, Sweden, Switzerland, Tajikistan, Turkey, Turkmenistan, Ukraine, United Kingdom, Uzbekistan. |
| **Eastern Mediterranean** | Afghanistan, Bahrain, Djibouti, Egypt, Iran, Iraq, Palestine, Jordan, Kuwait, Lebanon, Libya, Morocco, Oman, Pakistan, Qatar, Saudi Arabia, Somalia, Sudan, Syria, Tunisia, United Arab Emirates, Yemen |
| **Western Pacific Region** | Australia, Brunei, Cambodia, China, Cook Islands, Fiji, Japan, Kiribati, Laos, Malaysia, Marshall Islands, Micronesia, Mongolia, Nauru, New Zealand, Niue, Palau, Papua New Guinea, Philippines, Samoa, Singapore, Solomon Islands, South Korea, Taiwan, Tonga, Tuvalu, Vanuatu, Vietnam |
| **Income Classifications according to the World Bank Group calculated on Gross National Income (GNI) per capita in US Dollars** |  |
| **Low Income (LIC) (less than $1,145)** | Afghanistan, South Sudan, Burkina Faso, Liberia, Sudan, Burundi, Madagascar, Syria, Central African Republic, Malawi, Togo, Chad, Mali, Uganda, Congo, Mozambique, Yemen,  Eritrea, Niger, Ethiopia, Rwanda, Gambia, Sierra Leone, Guinea-Bissau, Somalia |
| **Low-Middle Income Country (LMIC) ($1,146-4,515)** | Angola, Jordan, Philippines, Algeria, India, Samoa, Bangladesh  Iran, Benin, Kenya, Senegal, Bhutan, Kiribati, Solomon Islands, Bolivia, Sri Lanka, Cabo Verde, Tanzania, Cambodia, Lebanon, Tajikistan, Cameroon, Lesotho, Timor-Leste, Comoros, Mauritania, Tunisia, Congo, Ukraine, Cote de Ivoire, Mongolia, Uzbekistan, Djibouti, Morocco, Vanuatu, Egypt, Myanmar, Vietnam, Eswatini, Nepal, Zambia, Ghana, Nicaragua, Zimbabwe, Guinea, Nigeria, Haiti ,Pakistan, Honduras, Papua New Guinea |
| **Upper-Middle Income Country (UMIC) ($4,516-14,005)** | Albania, Fiji, North Macedonia, Argentina, Gabon, Palau, Armenia, Georgia, Paraguay, Azerbaijan, Grenada, Peru, Belarus, Guatemala, Russia, Belize, Indonesia, Serbia, Bosnia and Herzegovina, Iraq, South Africa, Botswana, Jamaica, St.Lucia, Brazil, Kazakhstan, Bulgaria, Kosovo, Suriname, China, Libya, Thailand, Colombia, Malaysia, Tonga, Costa Rica, Maldives, Turkey, Cuba, Marshall Islands, Turkmenistan, Dominica, Mauritius, Tuvalu, Dominican Republic  Mexico, El Salvador, Moldova, Equatorial Guinea, Montenegro, Ecuador, Namibia |
| **High income countries (HIC) (greater than $14,005)** | American Samoa, Germany, Oman, Andorra, Gibraltar, Panama, Antigua and Barbuda, Greece, Poland, Aruba, Greenland, Portugal, Australia, Guam, Puerto Rico, Austria, Hong Kong, China, Qatar, Bahamas, Hungary, Romania, Bahrain, Iceland, San Marino, Barbados, Ireland, Saudi Arabia, Belgium, Seychelles, Bermuda, Israel, Singapore, British Virgin Islands, Brunei  Japan, Slovakia, Canada, South Korea, Slovenia, Cayman Islands, Kuwait, Spain, Channel Islands, Latvia, Chile, Liechtenstein, Croatia, Lithuania, Sweden, Luxembourg, Switzerland, Cyprus, China, Taiwan Czech Republic, Malta, Trinidad and Tobago, Denmark  Monaco, Turks and Caicos, Estonia, Nauru, United Arab Emirates, Faroe Islands, Netherlands, United Kingdom, Finland, United States, France, New Zealand, Uruguay, French Polynesia, Northern Mariana Islands, Guyana, Norway |

**Supplemental Table 3. Trial Characteristics According to Time Period (2007-2022)**

|  | **2007-2012**  **(n=35)** | **2013-2016**  **(n=36)** | **2017-2022**  **(n=53)** | **P-value** |
| --- | --- | --- | --- | --- |
|  | **N (%)** | **N (%)** | **N (%)** |  |
| **Phase** |  |  |  | .71 |
| I | 2 (5.7) | 4 (11.1) | 6 (11.3) |  |
| II | 7 (20) | 7 (19.4) | 7 (13.2) |  |
| III | 17 (48.6) | 17 (47.2) | 33 (62.3) |  |
| IV | 8 (22.9) | 7 (19.4) | 7 (13.2) |  |
| Missing |  |  |  |  |
| **Trial Length** |  |  |  | .13 |
| <2y | 12 (34.3) | 16 (44.4) | 18 (34) |  |
| 2-5y | 19 (54.3) | 15 (41.7) | 19 (35.8) |  |
| >5y | 4 (11.4) | 5 (13.9) | 16 (30.2) |  |
| **Sponsor Type** |  |  |  | .008 |
| Industry | 30 (85.7) | 36 (100) | 52 (98.1) |  |
| Academic | 5 (14.3) | 0 (0) | 1 (1.9) |  |
| **Hemophilia Type** |  |  |  | .26 |
| Hem A | 24 (68.6) | 20 (55.6) | 32 (60.4) |  |
| Hem B | 7 (20) | 12 (33.3) | 10 (18.9) |  |
| Hem A&B | 3 (8.6) | 4 (11.1) | 11 (20.8) |  |
| Missing | 1 (2.9) | 0 (0) | 0 (0) |  |
| **Severity** |  |  |  | .82 |
| All severities^c^ | 24 (68.6) | 21 (58.3) | 33 (62.3) |  |
| Moderate and Severe | 3 (8.6) | 7 (19.4) | 8 (15.1) |  |
| Severe | 4 (11.4) | 2 (5.6) | 4 (7.5) |  |
| Missing | 4 (11.4) | 6 (16.7) | 8 (15.1) |  |
| **Included inhibitor patients** | 5 (14.3) | 5 (13.9) | 16 (30.2) | .09 |
|  | **Mean (Standard Deviation)** | | |  |
| **Number of countries** | 6.3 (6.49) | 9.2 (5.48) | 8.1 (6) |  |
| **Number of Participants** | 61.6 (55.21) | 49.3 (38.38) | 65.3 (56.85) |  |
| **Average Participant Age** | 27.9 (10.3) | 24.7 (15.7) | 26.8 (12.5) |  |

**Supplemental Table 4. World Health Organization Income Differences in Observed, Expected, and Hemophilia Treatment Characteristics**

|  | **Low Income** | **Lower Middle Income** | **Upper Middle Income** | **Upper Income** |
| --- | --- | --- | --- | --- |
| **% of Trial Sites (2007-2022)** | 0% | 5.4% | 20.1% | 74.4% |
| **% of Factor Usage (2022)** | 0.3% | 6.0% | 39.0% | 55.0% |
| **Number of People with Hemophilia** | |  |  |  |
| Expected | 53,693 | 332,763 | 124,597 | 271,345 |
| **% of Expected** | 7% | 43% | 16% | 35% |
| **% of population** | 7% | 52% | 23% | 18% |
| **Per Capita Factor Usage (IU)** |  |  |  |  |
| Mean (Standard Deviation) | 0.05 (0.06) | 0.33 (0.61) | 1.79 (1.63) | 5.55 (3.64) |
| Factor Usage Range | 0.001-0.19 | 0.003-2.55 | 0.01-5.82 | 0.2-13.4 |
| **Clinical Trial Density per 1000 PwH** |  |  |  |  |
| Mean (Standard Deviation) | 0 (0) | 0.24 (1.4) | 2.67 (7.63) | 4.86 (7.95) |
| Range | 0 | 0-6 | 0-38 | 0-38 |

The number of identified people with hemophilia was not available across income categories.

1. Expected number of PwH among people counted in the WFH database
2. Population in all countries across the world

**Supplemental Table 5. Regional Differences in Observed, Expected, and Hemophilia Treatment Characteristics**

|  | **African** | **Americas** | **Eastern Mediterranean** | **Europe** | **Southeast Asia** | **Western Pacific** |
| --- | --- | --- | --- | --- | --- | --- |
| % of Trial Sites  (2007-2022) | 2.8% | 16.7% | 0.7% | 60.7% | 3.3% | 15.9% |
| % of Factor Usage (2022) | 1% | 40% | 5% | 41% | 4% | 9% |
| **Number of People with Hemophilia** | | |  |  |  |  |
| Identified | 8,003 | 57,954 | 35,576 | 65,632 | 36,648 | 53,333 |
| Expected | 94,130 | 100,085 | 75,318 | 81,981 | 213,492 | 198,738 |
| **% of people identified PwH** | 3.1% | 22.5% | 13.8% | 25.5% | 14.3% | 20.7% |
| **% of expected PwH^a^** | 12.3% | 13.1% | 9.9% | 10.7% | 38.0% | 26.0% |
| **Population by Region** | 16% | 17% | 11% | 12% | 37% | 7% |
| **Per Capita Factor Usage (IU per 1000 people with hemophilia)** | | | |  |  |  |
| Mean (Standard Deviation) | 0.28 (0.64) | 2.17 (2.85) | 1.17 (1.57) | 5.09 (3.61) | 0.17 (0.14) | 2.37 (3.19) |
| Range | 0.001-2.43 | 0.01-11.18 | 0.03-5.43 | 0-13.4 | 0.006-0.444 | 0.03-7.77 |
| **Clinical Trial Density** |  |  |  |  |  |  |
| Mean  (Standard Deviation) | 0.09 (0.61) | 0.48 (1.3) | 0.24 (1.1) | 6.9 (9.6) | 0.1 (0.31) | 1.9 (4.3) |
| Range | 0-4 | 0-7 | 0-5 | 0-38 | 0-1 | 0-16 |

1. Expected number of PwH among people counted in the WFH database
2. Population in all countries across the world
